# Modulation of Immunological Pathways in Autistic and Neurotypical Lymphoblastoid Cell Lines by the Enteric Microbiome Metabolite Propionic Acid

**DOI:** 10.3389/fimmu.2017.01670

**Published:** 2017-12-22

**Authors:** Richard E. Frye, Bistra Nankova, Sudeepa Bhattacharyya, Shannon Rose, Sirish C. Bennuri, Derrick F. MacFabe

**Affiliations:** ^1^Department of Pediatrics, University of Arkansas for Medical Sciences, Little Rock, AR, United States; ^2^Autism Research Program, Arkansas Children’s Research Institute, Little Rock, AR, United States; ^3^New York Medical College, Valhalla, NY, United States; ^4^Kilee Patchell-Evans Autism Research Group, Alberta Children’s Hospital Research Institute, Cumming School of Medicine, University of Calgary, Calgary, AB, Canada

**Keywords:** mitochondrial disease, autism, propionic acid, short-chain fatty acids, microbiome, inflammation, epigenetics, histone deacetylase inhibitor

## Abstract

Propionic acid (PPA) is a ubiquitous short-chain fatty acid which is a fermentation product of the enteric microbiome and present or added to many foods. While PPA has beneficial effects, it is also associated with human disorders, including autism spectrum disorders (ASDs). We previously demonstrated that PPA modulates mitochondrial dysfunction differentially in subsets of lymphoblastoid cell lines (LCLs) derived from patients with ASD. Specifically, PPA significantly increases mitochondrial function in LCLs that have mitochondrial dysfunction at baseline [individuals with autistic disorder with atypical mitochondrial function (AD-A) LCLs] as compared to ASD LCLs with normal mitochondrial function [individuals with autistic disorder with normal mitochondrial function (AD-N) LCLs] and control (CNT) LCLs. PPA at 1 mM was found to have a minimal effect on expression of immune genes in CNT and AD-N LCLs. However, as hypothesized, Panther analysis demonstrated that 1 mM PPA exposure at 24 or 48 h resulted in significant activation of the immune system genes in AD-A LCLs. When the effect of PPA on ASD LCLs were compared to the CNT LCLs, both ASD groups demonstrated immune pathway activation, although the AD-A LCLs demonstrate a wider activation of immune genes. Ingenuity Pathway Analysis identified several immune-related pathways as key Canonical Pathways that were differentially regulated, specifically human leukocyte antigen expression and immunoglobulin production genes were upregulated. These data demonstrate that the enteric microbiome metabolite PPA can evoke atypical immune activation in LCLs with an underlying abnormal metabolic state. As PPA, as well as enteric bacteria which produce PPA, have been implicated in a wide variety of diseases which have components of immune dysfunction, including ASD, diabetes, obesity, and inflammatory diseases, insight into this metabolic modulator may have wide applications for both health and disease.

## Introduction

The human microbiome represents a diverse ecosystem of microbes housed in the human body. Microbial cells outnumber the cells in the human body by a factor of 10 and microbial genes out number human genes by a factor of over 100 (1–3). There is a particular focus on the enteric (gut) microbiota since it represents about 99% of the human microbiome (4). The importance of the enteric microbiome in relation to human health and disease has been recognized since it appears to influence the immune system (5), metabolic processes (6), gene expression (7, 8), the nervous system (9, 10), and behavior (9, 10). Disruption of the enteric microbiome has been implicated in a wide range of human diseases including depression and anxiety (11), gastrointestinal disorders (12), inflammatory airway disease (13), diabetes (14–16), obesity (17, 18), atopic disease (5), and neurodegenerative conditions (19). The enteric microbiome may be particularly important early in life around the time of birth as it has been linked to early brain development and behavior (9, 10, 20) and disruption and/or treatments (i.e. early antibiotics) early in life can influence the development of childhood diseases, particularly atopic disease (9, 10).

The mechanism in which the enteric microbiome modulates particular effects on the host is not completely clear, although several mediators are potential vehicles for such influence. Such mediators include lipopolysaccharides, peptidoglycans, short-chain fatty acids (SCFAs), neurotransmitters and gaseous molecules (21–23). We are particularly interested in SCFAs because of their role as both mediators of physiology and mitochondrial fuels. SCFA are particularly intriguing as they are derived as a consequence of fermenting carbohydrates and some proteins, and also present naturally or as an additive in many foods, in particular wheat and dairy. Thus, dietary variations can have a larger influence on their production (19, 24, 25). Of the SCFAs, propionic acid (PPA) has been of key interest because it has several links to autism spectrum disorder (ASD), a disorder which affects as many as ~2% of children in the United States. What is intriguing about ASD is that the etiology is largely unknown but is strongly influenced by both genetic and environmental factors (26, 27).

The enteric microbiome is a major environmental factor that may contribute to the etiology of ASD (2, 9, 10, 28). First, several factors which may have a direct effect on health through disruption of the microbiome are associated with increased risk of developing ASD, including dietary alteration, environmental exposures that disrupt enteric microbiome bacteria content and diversity, being born by C-section delivery which reduces maternal transfer of enteric and vaginal bacteria, increased antibiotic use which can destroy key bacteria in the enteric microbiome, formula feeding and early hospitalization (2, 9, 28). Second, specific bacteria, such as *Clostridia* spp., a major SCFA producer, have been repeatedly reported to be overrepresented in the ASD microbiome (29, 30). Third, exposure to PPA has been demonstrated in several animal models to result in the development of ASD-like behaviors and physiological changes to the brain similar to those found in ASD are seen in adult rats acutely exposed to PPA (24, 25, 31) and in juvenile rats systematically exposed to PPA pre- and postnatally (32–34).

Although the mechanism by which PPA influences host function is still unclear, data from the animal model of PPA induced ASD demonstrates neuroinflammation and electrophysiological disturbances as well as disruptions in lipid, mitochondrial and redox metabolism (24, 25, 31). We have performed a series of studies to demonstrate that changes in mitochondrial metabolism similar to those found in the animal model exposed to PPA are also found in humans. For example, we found that the unique pattern of biomarkers of mitochondrial dysfunction found in the PPA rodent model was also found in a subset of children with ASD (28, 35, 36). We also demonstrated that PPA modulates mitochondrial respiration in lymphoblastoid cell lines (LCLs) derived from children with ASD differently than LCLs derived from age and gender matched typically developing control LCLs (37).

PPA also could induce changes in host physiology through modulation of the immune system. The animal models of PPA induced ASD behavior demonstrates neuroinflammation but inflammatory mediators induced by PPA in human ASD cells has not been investigated. In this study, we investigate whether PPA can differentially regulate immune genes using our LCL model of ASD. We have developed a cell line model of ASD in which LCLs derived from individuals with autistic disorder (AD) are classified into two groups: those with normal mitochondrial function (AD-N) and those with atypical mitochondrial function (AD-A) (38–40). The AD-A LCLs have respiratory rates approximately twice that of control and AD-N LCLs and are very sensitive to *in vitro* increases in reactive oxygen species (ROS) (38–40). We recently demonstrated that this atypical increase in mitochondrial function characteristic of AD-A LCLs was associated with more severe repetitive behaviors in the children from which these LCLs were derived (40). In this way, we believe that the AD-A LCLs may represent a more severe ASD phenotype. Given the connection between metabolism and immune system (41), we hypothesize that the AD-A LCLs will demonstrate a greater activation of immune genes with PPA exposure as compared to the control and AD-N LCLs.

## Materials and Methods

### LCLs and Culture Conditions

Lymphoblastoid cell lines were derived from white males diagnosed with AD chosen from pedigrees with at least other 1 affected male sibling (i.e., multiplex family) [mean (SD) age 7.3 (3.5) years]. These LCLs were obtained from the Autism Genetic Resource Exchange (Los Angeles, CA, USA) or the National Institutes of Mental Health (Bethesda, MD, USA) center for collaborative genomic studies on mental disorders. In our previous studies (37, 39, 40, 42–44), these LCLs where categorized into two different types of AD LCLs; ones with atypical mitochondrial respiration (AD-A) and those with normal respiration (AD-N). These metabolic groupings have been shown to be consistent and repeatable in our previous studies (37, 39, 40, 42–44). Eight pairs of AD-N and AD-A LCLs were age and gender matched to control LCLs. The sample size chosen was based on our previous studies. Control (CNT) LCLs were derived from healthy white male donors with no documented behavioral or neurological disorder and with no first degree relative suffering from any medical disorder that might involve mitochondrial dysfunction [mean (SD) age 7.5 (3.3) years]. CNT LCLs were obtained from Coriell Cell Repository (Camden, NJ, USA). Due to low availability of CNT LCLs which fit our criteria, a single CNT LCL line was paired with two AD LCL lines in one case (see Table 1). Also two AD-A LCLs were paired twice with AD-N LCLs. On average, cells were studied at passage 12, with a maximum passage of 15. Genomic stability is very high at this low passage number (45, 46). Cells were maintained in RPMI 1640 culture medium with 15% FBS and 1% penicillin/streptomycin (Invitrogen, Grand Island, NY, USA) in a humidified incubator at 37°C with 5% CO_2_.

**Table 1:** Lymphoblastoid cell lines used in this study.

Controls			AD-N subgroup			AD-A subgroup		
Cell ID	Source	Age (years)	Cell ID	Source	Age (years)	Cell ID	Source	Age (years)
GM09659	Coriell	4	04C24363	NIMH	4	1393306	AGRE	3
GM17255	Coriell	6	02C10054	NIMH	6	01C08594	NIMH	7
GM16007	Coriell	12	05C38988	NIMH	12	1165302	AGRE	13
GM18054	Coriell	5	03C15992	NIMH	5	01C08495	NIMH	4
GM11626	Coriell	13	008404	AGRE	13	1165302	AGRE	13
GM09642	Coriell	7	01C08367	NIMH	7	01C08594	NIMH	7
GM09642	Coriell	7	04C27439	NIMH	7	02C09713	NIMH	7
GM09380	Coriell	6	01C08022	NIMH	5	01C08495	NIMH	4

*Three types of cell lines were used with two types of autistic disorder (AD) cell lines, characterized in our previous studies, and one type of control cell line*.

### PPA Exposure

Each group of LCLs were cultured with PPA 1 mM for 24 or 48 h or left untreated (0 mM). This concentration was selected because it provided optimal metabolic activation in our previous studies (37). The sodium propionate was buffered with sodium bicarbonate in the culture medium to prevent changes in pH which could cause changes in influx of PPA (47). As PPA is mostly disassociated at physiological pH, the effects of the PPA treatment are most likely a combination of both PPA and propionate.

### Expression Studies

Total RNA samples from each LCL group were pooled together and after DNase treatment and purified using RNeasy Mini Kit (Qiagen Sciences, MD, USA) as described in our previous studies (48). The cDNA synthesis and microarray analyses were performed at Keck Affymetrix GeneChip Resource at Yale, New Haven, CT, USA (NIH Neuroscience Microarray Consortium) as previously described (48).

### Analytic Approach

Analysis of variance was conducted between the exposure conditions and different cell types. Genes showing expression of at least ≥2.0-fold were exported for functional annotation to several pathway analysis packages including Ingenuity Pathway Analysis (IPA) and Panther software. For the initial comparison of the effect of PPA for each exposure time on a particular LCL type, the statistical significance of the comparison was not considered as there was only an *N* of 1 for each example. When the ASD LCL types were compared to controls, the two PPA exposure times were combined and the genes selected not only showed a difference in expression of at least ≥2.0-fold but also a *p* < 0.05.

## Results

### The Effect of PPA on Gene Expression for Each LCL Type

The change in gene expression resulting from 1 mM exposure to PPA for 24 and 48 h was determined for each LCL type separately. Table S1 in Supplementary Material demonstrates the number of genes up- and downregulated more than 2.0-fold for each LCL type.

The CNT LCLs demonstrated no upregulation or downregulation of known genes with 24 h PPA exposure and only one gene upregulated and downregulated with 48 h PPA exposure. Only the downregulated gene was associated with immune function. Panther analysis demonstrated no overrepresentation of immune genes associated with PPA exposure in CNT LCLs.

Exposure of AD-N LCLs to PPA for 24 h demonstrated no upregulated genes and downregulation of several immune genes including two major histocompatibility complex genes. Exposure of AD-N LCLs to PPA for 48 h demonstrated upregulation of two microRNA genes not known to be involved in immune function and downregulation of the gene for complement C4B. Panther analysis demonstrated overrepresentation of genes associated with major histocompatibility complex antigen with 24 h PPA exposure in AD-N LCLs (see Table 2).

**Table 2:** Panther overrepresentation analysis of genes significantly upregulated and downregulated with 24 and 48 h PPA exposure.

	# of genes	Enrichment	*p*-Value
**AD-N downregulated 24 h**			
Protein class			
Major histocompatibility complex antigen	2	91.31	<0.05
**AD-A upregulated 24 and 48 h**			
Biological process			
Immune response	6	20.87	<0.0001
Response to stimulus	7	5.83	<0.01
Cellular component			
Immunoglobulin complex	2	54.91	<0.05
Extracellular space	6	26.32	<0.0001
Extracellular region	6	19.08	<0.0001
Protein class			
Immunoglobulin	4	99.3	<0.0001
Defense-immune protein	4	19.78	<0.01
Molecular function			
Antigen binding	4	44.66	<0.01
Biological processes			
Immunoglobulin production	4	79.78	<0.01
Production of mediator of immune response	4	69.14	<0.01
Immune response	7	9.83	<0.01

Exposure of AD-A LCLs to PPA for 24 or 48 h demonstrated upregulation of several genes related to immune function, particularly several genes associated with immunoglobulin production and one gene related to activation of proinflammatory caspases. Downregulation of the gene for complement C4B was found for 24 h exposure and no genes were downregulated for 48 h exposure. Panther analysis demonstrated overrepresentation of many immune processes and proteins as result of PPA exposure to AD-A LCLs for 24 and 48 h, demonstrating that PPA did significantly activate immune processes for AD-A LCLs (Table 2).

### Comparison of PPA Effect on ASD LCLs as Compared to Control LCLs

**Figure 1:**
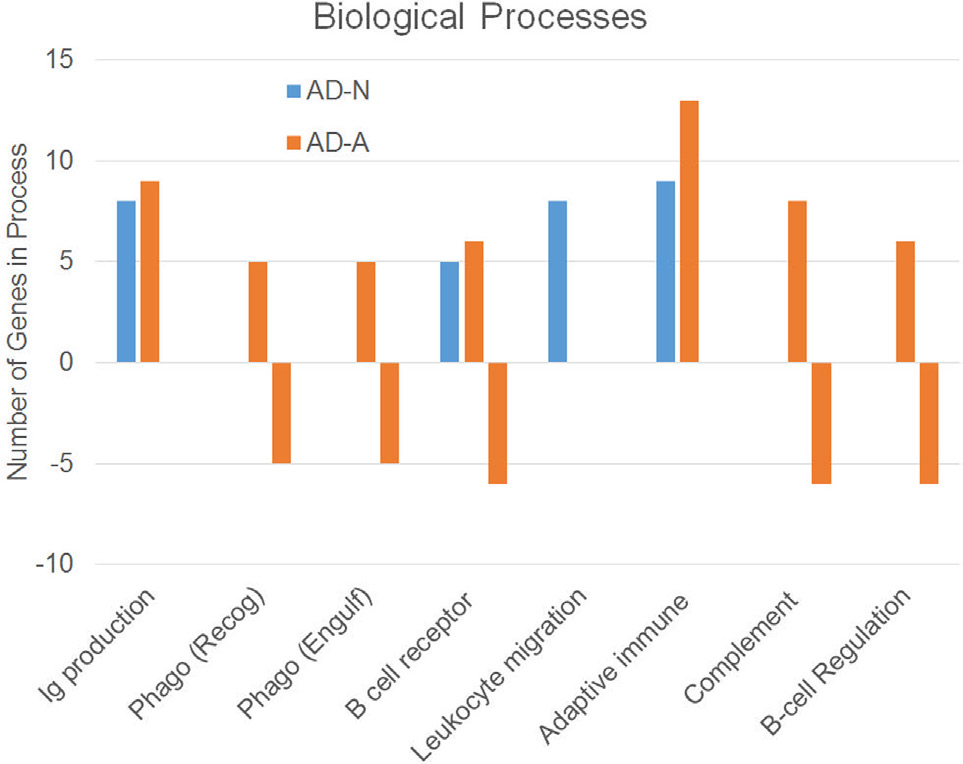
Biological processes associated with an increase or decrease in gene expression resulting from propionic acid exposure to autism cell lines as compared to control cell lines.

To better understand how PPA exposure affects ASD LCLs differently than control LCLs, gene expression was compared between CNT LCLs and each ASD LCL group independently. Both the 24- and 48-h PPA exposure data was combined since the previous analysis demonstrated little difference between the changes in gene expression with these two different exposure durations. Table S2 in Supplementary Material outlines the genes that were upregulated or downregulated with PPA exposure for each ASD LCL group as compared to CNT LCLs. Table 3 demonstrates the biological processes identified by the differential gene expression for AD-N and AD-A LCLs as compared to CNT LCLs. The major processes identified are also represented in Figure 1. Biological process was the only Panther analysis used as it was the most robust for representing the difference in pathway activation.

**Table 3:** Biological processes panther overrepresentation analysis of genes differentially expressed in autism cell lines as compared to control cell lines.

	AD-N LCLs		AD-A LCLs	
	Up	Down	Up	Down
Immunoglobulin production	8	0	9	0
Mediator of immune response	8	0	9	0
Immune system process	22	0	20	0
Phagocytosis (recognition)	0	0	5	5
Phagocytosis	0	0	9	6
Phagocytosis (engulfment)	0	0	5	5
Plasma membrane invagination	0	0	5	5
Membrane invagination	0	0	5	5
B cell receptor signaling pathway	5	0	6	6
Antigen receptor-mediated signaling	8	0	8	8
Activating cell surface receptor	10	0	12	10
Regulating cell surface receptor	10	0	12	10
Response-regulating signaling	10	0	12	10
Response-activating signaling	10	0	12	10
Activation of immune response	10	0	12	10
Regulation of response	12	0	13	10
Leukocyte migration	8	0	0	0
Cell migration	13	0	0	0
Cell motility	13	0	0	0
Locomotion	13	0	0	0
Subcellular component movement	14	0	0	0
Localization of cell	13	0	0	0
Adaptive immune response	9	0	13	0
Immune response	19	6	20	0
Complement activation, classical pathway	0	0	8	6
Complement activation	0	0	8	6
Protein activation cascade	0	0	9	6
Humoral immune response	0	0	9	6
Mediated by immunoglobulin	0	0	9	6
Immunoglobulin mediated	0	0	9	6
B cell-mediated immunity	0	0	9	6
Receptor recombination	0	0	9	7
Lymphocyte mediated	0	0	9	8
Leukocyte mediated	0	0	12	8
Regulation of B cell activation	0	0	6	6

*Numbers represent number of genes associated with the identified biological process*.

This analysis suggests that both the AD-N and AD-A LCLs demonstrate change in immune genes as compared to CNT LCLs. Both AD-A and AD-N LCLs demonstrate an upregulation in genes associated with immunoglobulin production and adaptive immune responses without any downregulation in genes involved in these processes. AD-A LCLs demonstrate both upregulation and downregulation of genes involved in a wider variety of immune responses as compared to AD-N LCLs, including phagocytosis, complement system activation, B cell regulation, and B cell receptors. This suggests that AD-A LCLs may have a wider network of immune genes activated as compared to AD-N LCLs as well as CNT LCLs.

Table 4 represents the top canonical pathways (*p* < 0.01) identified by IPA for the comparison between the AD-A and CNT LCLs. As we see, many of these processes are involved in immune activation and immune disorders. IPA also identified the top upstream regulators as RUNX3, ONECUT1, SNAI2, STAT5A, and TCF7. Interestingly, as will be discussed below, these genes are regulatory of both developmental and immune processes.

**Table 4:** Top canonical pathways identified using Ingenuity Pathway Analysis (IPA).

B cell development
T helper cell differentiation
Primary immunodeficiency signaling
Graft-versus-host disease signaling
Calcium-induced T lymphocyte apoptosis
IL-4 signaling
Altered T cell and B cell signaling in rheumatoid arthritis
Antigen presentation pathway

## Discussion

In this study, we examined the effect of PPA, a SCFA produced by enteric bacteria that are overrepresented in the ASD gut, on transformed B cells (LCLs) derived from children with ASD as well as controls. We examined two types of LCLs derived from children with ASD, those with mitochondrial dysfunction (AD-A) and those found to have mitochondrial function similar to controls (AD-N). We hypothesized that PPA would activate immune pathways in ASD LCLs since the PPA animal model of ASD demonstrates neuroinflammation and immune activation, including increased GFAP immunoreactivity in the hippocampus, increased activation of microglia, and increased interleukin (IL)-6 (24, 25, 31). We further hypothesized that the AD-A LCLs would have a greater enhancement of immune pathways since this is a more severe ASD phenotype and since optimal mitochondrial function is required for appropriate immune function and response (41).

Exposure to PPA for either 24 or 48 h resulted in upregulation in genes associated with immune system activation in AD-A LCLs, particularly genes involved in immunoglobulin production. This effect was not seen in CNT or AD-N LCLs. In fact, there was a decrease in major histocompatibility complex antigen genes in AD-N LCLs exposed to PPA for 24 h. We then compared the effect of PPA on ASD LCLs as compared to the effect of PPA on CNT LCLs. We found that both the AD-N and AD-A LCLs demonstrated changes in gene expression as compared to the control LCLs with a significant change in genes related to immune pathways almost exclusively. Although the AD-N LCLs demonstrated activation of immune pathways, the AD-A LCLs demonstrated a wider range of genes and processes involved in immune pathways. In addition, IPA analysis of AD-A LCL gene expression changes identified canonical pathways almost exclusively related to immune function.

Several of the genes identified by the IPA analysis are involved in regulation of the immune system and may be linked to ASD. Several genes are linked to regulation of T cells. TCF7 is a T lymphocyte-specific enhancer of the CD3-Epsilon T cell antigen receptor complex. Interestingly TCF7 expression may be regulated by beta-catenin (49). This is intriguing since beta-catenin has been shown to be dysregulated in an animal model of ASD (50). STAT5 is induced in response to T cell activation with cytokines, most notably IL-2, and is believed to be involved in the effect of IL-2 in the immune response and may be involved in the suppression of IL-3 production. This is interesting as IL-2 is produced by neurons and astrocytes, is important in brain development and normal brain physiology and has been implicated in neurodegenerative disease, cognitive dysfunction and has been linked to ASD (51). RUNX3 is also important in immune system function as well as neuronal development. RUNX3 is essential during thymopoiesis where it modulates the development of CD8 T cells, thus having an important role in immune system development through lineage specification (52). Interestingly, RUNX3 is involved in the TNF-beta signaling cascade (53), a cytokine whose dysregulation has been correlated with ASD severity (51). RUNX3 appears to have an important role in the development of proprioceptive afferent neurons in mice, resulting in ataxia (54), a neurological finding that is not uncommon in ASD. Other genes identified are related to B cell function. SNAI2 is an evolutionarily conserved zinc finger transcription factor which plays an important role in prenatal fetal development, most notably the development of neural crest-derived cells and adipocytes (55). SNAI2 is also involved in regulation of B cells and can promote the aberrant survival and malignant transformation of mammalian pro-B cells otherwise slated for apoptotic death (56) and has antiapoptotic effects (57).

In conclusion, ASD is being recognized as having a very strong immune component to its etiology (58). Several models of ASD demonstrate immune dysregulation, including prenatal exposure to immune challenges (59, 60). In fact two animal models have been developed to parallel prenatal exposure to autoantibodies (61), including fetal brain antibodies (62) and antibodies to the folate transporter (63, 64). The microbiome is being recognized as important in the etiology of neurodevelopmental disorders (9, 10), potentially through modulation of the immune system (65) through enteric metabolites (65) including SCFAs like PPA (24, 25, 31). It is important to note the effects of SCFA on gene expression and inflammation are complex, and include histone deacetylase activity, activation of free fatty acid G-coupled receptor and mitochondrial inflammatory signaling cascades, which may or may not be mutually reinforcing. Furthermore, we do not yet know if the effects found in our LCL model also occur in patients, as many effects of SCFA, in particular PPA and butyrate, are dose and tissue dependent, and have different effects at key developmental time periods (9, 10, 24, 31, 48, 66, 67). Nonetheless, this study provides insight into the mechanism in which the microbiome may influence the immune system to result in disease and demonstrates the predisposition of certain cells to be sensitive to microbiome metabolites. It also may lead to further reevaluation of the widespread use of PPA in agriculture and the food industry (24, 31). Certainly, further research is needed in this area to better define the role of the microbiome and microbial metabolites in immune modulation and disease.

## Author Contributions

The conception and design of the work was agreed upon by all authors as was the drafting and final approval of the manuscript. BN, SR, and SB were involved in laboratory analysis. SB was involved in data analysis. RF, DM, SB, SR, and SB were involved in interpretation of data.

## Conflict of Interest Statement

The authors declare that the research was conducted in the absence of any commercial or financial relationships that could be construed as a potential conflict of interest.
