# Supplementary material for: Modulation of Immunological Pathways in Autistic and Neurotypical Lymphoblastoid Cell Lines by the Enteric Microbiome Metabolite Propionic Acid
Source: Front Immunol. 2017 Dec 22;8:1670. doi: 10.3389/fimmu.2017.01670 (PMC5744079; doi:10.3389/fimmu.2017.01670)
Supplement: Supplementary file 1 [file Table_1.DOCX]

| Table S1. Identified genes increased or decreased at least 2.0 fold for each lymphoblastoid cell line type for two incubation time (24hrs and 48hrs) of propionic acid as compared to no exposure to propionic acid. | | | | | | |
| --- | --- | --- | --- | --- | --- | --- |
|  | Control | | AD-N | | AD-A | |
|  | 24 hr | 48 hr | 24 hr | 48 hr | 24 hr | 48 hr |
| **Up** | *None* | OR2J2 | *None* | MIR548M  MIR548X | CREB5  IGH  IGKC  IGHV2-70  IGKV2-28  IGKV2D-28  IGKV2-24  IGKV2D-26  LEPREL1  NLRP2  RP11-567J20.3  RAB31 | CREB5  IGH  IGKC  IGHD3-16  IGHV2-70  IGKV2-28  IGKV2D-28  IGKV2-24  IGKV2D-24  IGKV2D-26  LEPREL1  NLRP2  RP11-567J20.3 |
|  |  |  |  |  |  |  |
| **Down** | *None* | IGHD3-22 | C4B  HLA-B  HLA-DQB1  SLC25A5  RP11-124N14.3  RP11-1079K10.4  TAB3-AS2  HNRNPA1P44  NPIPB4  NPIPB3  NPIPB5  KMT2C  KPNB1  PCNA-AS1  DNAJA1  MALAT1  REPIN1  MIR3661  MIR1184-1 | C4B  OR2J2 | C4B  OR2J2 | *None* |

| Table S2. Identified genes increased or decreased at least 2.0 fold and significant at the p<0.05 level for each autism lymphoblastoid cell line type as compared to the control cell lines for two propionic acid incubation times combined (24hrs and 48hrs). | | | | |
| --- | --- | --- | --- | --- |
|  | AD-N | | AD-A | |
| **Up** | *IGHD3-10*  *IGKV2D-40*  *IGKV2-40*  *IGLV6-57*  *AL928768.3*  *IGH; IGHA1*  *IGLV3-1*  *IGKV2-28*  *IGKC*  *IGKV1D-37*  *IGKC*  *IGKV1D-16*  *IGKV2D-28*  *IGKV1-6*  *LEPREL1*  *IGHV1-8*  *IGKV1D-42*  *CREB5*  *PLS3*  *IGKV1D-33*  *NAPSB*  *UCHL1*  *TUBB2B*  *ADAM23*  *RP1-241P17.1*  *MUC13*  *IGKV3D-15*  *NAPSB*  *MNDA*  *IGKV1-9*  *IGHV3-9*  *SCIMP*  *IGKV1-17*  *IGKC*  *ITGAX*  *IGHV7-81*  *SIRPG*  *GCNT1*  *HLA-DRB5*  *RAB31*  *CD226*  *PRKCH*  *NPNT*  *IGKV2-24*  *BLK*  *NAALADL2-AS2*  *SLC12A8*  *SLC23A2*  *HCK*  *SMIM14*  *IGKC*  *GSTT1*  *JUP*  *TNFSF14*  *BLK*  *TUBB2A*  *FHOD3*  *DNMBP*  *CDKL5* | | IGHD3-10  AL928768.3  IGHV1-18  IGLV3-1  IGH; IGHA1  IGKV1-17  IGLV4-69  IGKV1D-16  IGKV1D-42  IGKV1-6  IGKV1-5  IGKV1D-37  IGKV2-28  IGKC  IGHV1-8  IGKC  TUBB2B  TUBB2B  IGKV2D-28  IGKV1-9  PRKCH  IGHV5-51  IGKC  MNDA  IGKV1-8  UBASH3B  IGKV1D-33  UCHL1  GPR128  TUBB2A  SLC12A8  PTPRS  LEPREL1  JUP  ADAM23  HLA-DRB5  IGHV7-81  HCK  SCIMP  NETO1  GATM  GSTT1  SHISA3  FHL1  HDGFRP3  KAL1  BAIAP2L1  ANO5  F13A1  PTPN14  HLA-DQB1  TMOD1  DXO  APBB2  TBC1D4 | |
|  |  |  |  |  |
| **Down** | FILIP1  C14orf105  TDRD12  CD96  LZTFL1  EFNA5  SLC10A2  TMEM2  GALM  EPB41L3  PTPRK  ABCC9  IL12RB2  CD38  T  FFAR2  IGKV3-11  GBP5  PRRX1  IGHV1-58  PSTPIP2  IGHV3-21  IGLV3-25  IGHV2-70  IGKV2-29  GTSF1  IGHD3-3  IGKV5-2 | | HNRNPLL  CDH1  ZC4H2  CDC42BPA  MAN1A1  EPS8  MGST1  TDRD12  GPR125  CMTM7  TCN2  CACNB2  GALM  ROBO1  IGLL5  PITPNC1  RBPMS  RNU5A-8P  HIPK2  RORA  PTPN13  HIPK2  EGOT  CD96  IGHV6-1  PTPN13  HLA-DQA1  HLA-DQA1  APOL1  T  RASSF6  IGLC7  LZTFL1  FFAR2  SUCNR1  DSC3  PRRX1  IL12RB2  IGHV1-58  IGKV4-1  TBXAS1  IGHM  CD38  IGLV6-57  IGHV3-21  IGKV3-11  GTSF1  IGHV3-9  IGKV2-29  IGKV5-2  IGHD3-3 | |
